# New Appliances and Things Medical

**Published:** 1908-05-16

**Authors:** 


### NEW APPLIANCES AND THINGS MEDICAL.

### A "NON-SLIPPING" CARPET.

We have received from Mr. A. Ambrose, of The Croft, Loughton, Essex, a small "non-slipping" carpet, which should prove of service in the wards of hospitals and in other places where the floors are of necessity polished and slippery. The under surface is coated with an impervious semi-adhesive substance, which entirely prevents lateral movement of the mat upon even the most highly polished floor. At the same time, the mat can be raised from the floor without the least difficulty, and it can be beaten or washed or otherwise cleaned without impairment of its special qualities. The small specimen carpet which was sent to us by the inventor has been in use now for several months and has not deteriorated in any way; and we cannot find that the special preparation of the under surface detracts from the appearance of the polished floor boards with which it has been in contact. This invention would prevent those accidents which occasionally happen in hospitals and houses owing to the slipping of mats under the feet of persons walking in a hurry or carrying bulky articles. Polished floors are now almost universal in hospital wards, and the substitution of mats prepared in this manner for the ordinary small carpets would reduce the risk of accidents due to slipping, and would abolish one of the objections to the use of hearth-rugs in wards provided with fireplaces or stoves.

### CARABANA WATER.

This is a very active purgative water. It is exceptionally rich in sodium sulphate, containing nearly 230 grammes of this salt per litre. Magnesium sulphate is present in the proportion of 6 grammes per litre, and the chlorides of sodium, magnesium and calcium in smaller quantities. There is 1.5 per cent. of sodium sulphide and nearly .4 per cent. of sodium phosphate. Considering the high percentage of saline purgatives which this water contains, its taste is not particularly unpleasant, while its concentration renders it very convenient for travelling. We have found it rapid and energetic in action, and quite free from disagreeable effects. Carabaña should prove of much use in all hepatic and intestinal disorders in which a powerful saline aperient is indicated, and in congestive conditions of various kinds. The agents in this country are Findlater and Co., Findlater's Corner, London Bridge, S.E.

